# CD33 Expression on Peripheral Blood Monocytes Predicts Efficacy of Anti-PD-1 Immunotherapy Against Non-Small Cell Lung Cancer

**DOI:** 10.3389/fimmu.2022.842653

**Published:** 2022-04-14

**Authors:** Claire Olingy, Ahmad Alimadadi, Daniel J. Araujo, David Barry, Norma A. Gutierrez, Max Hardy Werbin, Edurne Arriola, Sandip Pravin Patel, Christian H. Ottensmeier, Huy Q. Dinh, Catherine C. Hedrick

**Affiliations:** ^1^ Center for Cancer Immunotherapy, La Jolla Institute for Immunology, La Jolla, CA, United States; ^2^ Cancer Research Program, Institut Hospital del Mar d’Investigacions Mèdiques, Barcelona, Spain; ^3^ Medical Oncology Department, Hospital del Mar-Centro de Investigación Biomédica en Red de Oncología (CIBERONC), Barcelona, Spain; ^4^ Moores Cancer Center, University of California, San Diego, La Jolla, CA, United States; ^5^ Institute of Translational Medicine, Department of Molecular & Clinical Cancer Medicine, University of Liverpool, Liverpool, United Kingdom; ^6^ McArdle Laboratory for Cancer Research, University of Wisconsin-Madison, Madison, WI, United States

**Keywords:** CD33, monocytes, immunotherapy, non-small cell lung cancer, immunosuppression

## Abstract

Non-small cell lung carcinoma (NSCLC) is the leading cause of cancer-related deaths globally. Immune checkpoint blockade (ICB) has transformed cancer medicine, with anti-programmed cell death protein 1 (anti-PD-1) therapy now well-utilized for treating NSCLC. Still, not all patients with NSCLC respond positively to anti-PD-1 therapy, and some patients acquire resistance to treatment. There remains an urgent need to find markers predictive of anti-PD-1 responsiveness. To this end, we performed mass cytometry on peripheral blood mononuclear cells from 26 patients with NSCLC during anti-PD-1 treatment. Patients who responded to anti-PD-1 ICB displayed significantly higher levels of antigen-presenting myeloid cells, including CD9^+^ nonclassical monocytes, and CD33^hi^ classical monocytes. Using matched pre-post treatment samples, we found that the baseline pre-treatment frequencies of CD33^hi^ monocytes predicted patient responsiveness to anti-PD-1 therapy. Moreover, some of these classical and nonclassical monocyte subsets were associated with reduced immunosuppression by T regulatory (CD4^+^FOXP3^+^CD25^+^) cells in the same patients. Our use of machine learning corroborated the association of specific monocyte markers with responsiveness to ICB. Our work provides a high-dimensional profile of monocytes in NSCLC and links CD33 expression on monocytes with anti-PD-1 effectiveness in patients with NSCLC.

## Introduction

Lung cancer is the leading cause of death worldwide, with non-small cell lung cancer (NSCLC) accounting for approximately 85% of all newly diagnosed cases of the disease (1). Signaling between Programmed Cell Death Protein 1 (PD-1) on CD8^+^ T cells and its cognate ligand PD-L1 on other immune cells or cancer cells drives cancer progression (2, 3). Immune checkpoint blockade (ICB) therapy targeted at inhibiting the PD-L1:PD-1 signaling axis is beneficial for patients with a variety of cancers including NSCLC (2, 3). Still, many patients with NSCLC are either non-responsive (2, 4) or eventually become resistant to anti-PD-1 treatment (5). There is thus an unmet need to pinpoint immunological markers that accurately predict ICB responsiveness.

Monocytes have recently emerged as attractive targets for identifying immunological phenotypes associated with differential responses to anti-PD-1 therapy. Monocytes are mononuclear phagocytes that rapidly respond to inflammatory signals and act to coordinate many aspects of innate and adaptive immunity (6). Peripheral blood monocytes originate in the bone marrow and fall into three major categories: the classical (C14^+^CD16^-^), intermediate (C14^+^CD16^+^), and nonclassical (C14^-^CD16^+^) subsets (6). Once they extravasate into tissues, monocytes differentiate into a spectrum of cell types that includes macrophages and certain types of dendritic cells. Monocyte-derived macrophages and dendritic cells themselves induce specific T cell programs by releasing cytokines, upregulating co-stimulatory and co-inhibitory molecules, and cross-presenting antigens (7).

Subsets of monocytes and monocyte-derived cells exhibit a dynamic range of phenotypes and functions in response to solid tumors. Classical CD14^+^CCR2^+^ monocytes infiltrate neoplasms and contribute to the majority of myeloid cells within the tumor microenvironment (8, 9), but they are eventually reprogrammed into pro-tumoral monocyte-derived cells by the cancer niche (10). In contrast, nonclassical (CD16^+^CD56^-^) monocytes exhibit both tumor-killing and anti-metastatic properties (11, 12). Certain subpopulations of monocytes and monocyte-derived cells are also differentially associated with responses to anti-PD-1 ICB (13–15), and PD-L1 expression on monocytes spurs cancer progression (16, 17). Nevertheless, our understanding of the relationship between monocyte heterogeneity and ICB responsiveness remains incomplete.

In order to identify novel relationships between monocyte markers and positive responses to anti-PD-1 therapy, we used cytometry by time-of-flight (CyTOF) to explore monocyte heterogeneity in two cohorts of NSCLC patients receiving anti-PD-1 ICB treatment. We identified CD9^+^ nonclassical monocytes and CD33^hi^ classical monocytes as two populations that are positively correlated with ICB responsiveness. The use of machine learning recapitulates the importance of CD33 expression on monocytes during anti-PD-1 therapy. Our results link specific monocyte subsets, and particularly CD33^hi^ classical monocytes, with the effectiveness of anti-PD-1 treatment for NSCLC.

## Materials and Methods

### Human Samples

We obtained peripheral blood mononuclear cells (PBMCs) from patients with NSCLC from two clinical sites: University of California San Diego in San Diego (UCSD), and Institut Hospital del Mar d’Investigacions Mèdiques (IMIM) in Barcelona. Patient samples were collected under a UCSD ImmunoScape Immune Monitoring Protocol (HRPP# 150348; PI: S. Patel) and a CEIC-PSMAR-approved protocol at IMIM (2017/7174; PI: E. Arriola). All studies were approved by the IRB at La Jolla Institute for Immunology. The average patient age was 69.3 years, and 65% percent of the subjects were male. Approximately 16% of patients had advanced NSCLC (stage IV). All patients were treated with either Pembrolizumab or Nivolumab as the sole therapy. Patient characteristics are shown in **Supplementary Table 1**. Response status was assessed after 6 months of treatment, with responders defined as patients who showed clinical benefit (decrease in tumor size or stable disease) for at least the first 6 months of treatment. Non-responders include patients who progressed or discontinued therapy (due to clinical deterioration or death) within the first 6 months of treatment. Blood from melanoma patients was obtained from the Biospecimen Repository Core Facility at The University of Kansas Cancer Center.

### CyTOF Mass Cytometry

Directly-conjugated antibodies were purchased from Fluidigm and purified antibodies were ordered from the companies listed below. Conjugations were performed with the Maxpar X8 Multi-Metal Labeling Kit (Fluidigm). Cells were stained with 5mM Cisplatin (Fluidigm; live/dead), a metal-conjugated antibody cocktail, and 125nM Ir-Intercalator (Fluidigm). Cells were then resuspended at 1×10^6^cells/ml in 0.1×EQ™ Four Element Calibration Beads (Fluidigm), and the acquisition was performed on the CyTOF Helios2 (Fluidigm). Batch effects and signal drifting were minimized by tuning every 6hrs during long runs. To test for consistency between CyTOF acquisitions, a single healthy donor patient sample was included in each run. 100ml of heparinized blood was drawn from a healthy control donor, PBMCs were isolated and aliquots were frozen (90%FBS+10%DMSO) and stored at -80°C until use. Between every CyTOF run, one vial was taken for staining and acquisition.

### CyTOF Data Clustering and Analysis

CyTOF data were processed using a CyTOF workflow, and analyses were implemented using the *CATALYST* package in R (18). CyTOF data were arcsinh (inverse hyperbolic sine) transformed with a cofactor of 5, and batch-corrected using the quantile normalization method for the pooled distribution of each batch (a pair of sample and spike-in control) implemented in the function *normalizeBatch* from the *cydar* Bioconductor package (19). Self-Organizing-Map-based method *FlowSOM* (20) was used for clustering of cells. Visualization and high-dimensional reduction were performed using the UMAP method (21). Differentially expressed marker analysis was carried out using linear modeling, *limma* (22), and differential cell population abundance was determined by a Mann-Whitney test (*p* < 0.05). The Destiny package was used to perform diffusion map pseudotime for trajectory analyses (23).

### Flow Cytometry

To analyze peripheral immune cell populations, 0.5ml heparinized blood samples from additional healthy volunteers, NSCLC patients from UCSD and IMIM, and melanoma patients were stained and analyzed by flow cytometry. The following antibodies were used: Live/Dead Fixable Yellow (Invitrogen), CD14 (63D3), CD163 (GHl/61), CD274 (29E.2A3), CXCR3 (6025H7), CD33 (WM53), CD16 (3G8), Slan (M-DC8), CD273 (24F.10C12), CD9 (HI9a), HLADR (L243), CD64 (10.1), CD1c (L161), CD56 (5.1H11), CD3 (OKT3), CD19 (HIB19), CD66b (G10F5), Siglec-8 (7C9), CD279 (EH12.2H7), CCR7 (G043H7), CD45RA (HI100), CD4 (RPA-T4), CD45RO (UCHL1), CD69 (FN50), CD25 (BC96), CD127 (A019D5). Samples were acquired on a BD LSRII. Frequencies of monocyte subsets were correlated with regulatory T cell subset frequencies in the blood of the same patients.

### Data From the Cancer Genome Atlas Program

TCGA survival data was linked to CD33 and CD9 expression in patients with lung adenocarcinoma, lung squamous cell carcinoma, and skin cutaneous melanoma by downloading data from http://www.oncolnc.org/ (24). Survival data up to 1500 days for patients with skin cutaneous melanoma, lung adenocarcinoma, and lung squamous cell carcinoma were used for survival analysis. Log-rank tests were performed using the *survival* package (25) and Kaplan-Meier curves were generated using the *survminer* package (26).

### Monocyte:T Cell Interactions in the Tumor Microenvironment

We performed ligand:receptor interaction analysis of T cells and monocytes in recently published single-cell RNA-sequencing data from normal lung tissue and adenocarcinoma tissue (27). We utilized the *CellChat* (28) algorithm to assess interactions between monocytes and regulatory T cells. To perform this analysis, we used normalized data and followed the official CellChat workflow for comparative analysis of multiple conditions.

## Results

### High-Dimensional Profiling of Peripheral Immune Cells From Patients With NSCLC

**Figure 1:**
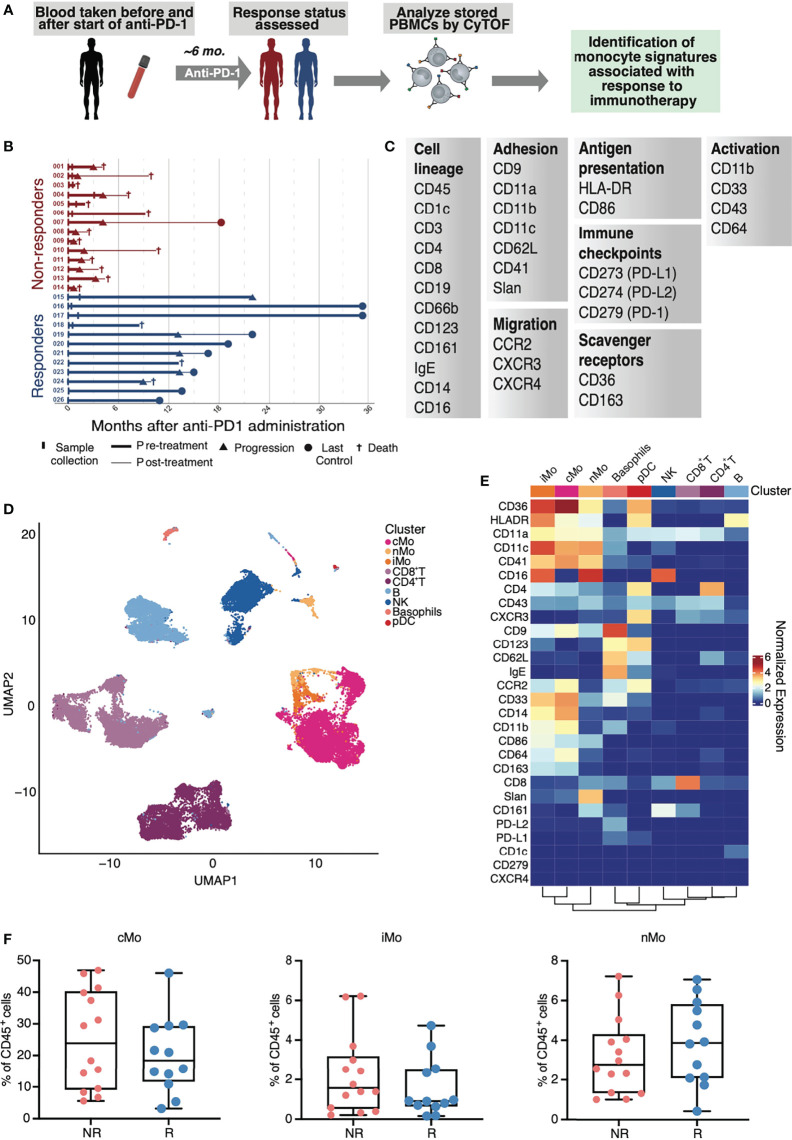
Study design and mass cytometry-based analysis of PBMCs from patients with NSCLC. **(A)** Study design of sample collection from patients with NSCLC and treated with anti-PD-1. **(B)** Visual summary of patient cancer progression and survival information for NSCLC cohorts. **(C)** Markers used in a monocyte-focused CyTOF mass cytometry panel used in this study. **(D)** UMAP of identified CD45^+^ immune cells in PBMCs from CyTOF mass cytometry analysis of NSCLC subjects. **(E)** Heat map of inverse hyperbolic sine (arcsinh)-normalized marker expression for each immune cell cluster identified in **(D)**. **(F)** Total frequencies of classical (cMo), intermediate (iMo) and nonclassical monocytes (nMo) in non-responders (NR) and responders (R) from CyTOF analysis of CD45^+^ cells in NSCLC subjects; each dot represents an individual patient.

To assess monocyte heterogeneity in response to anti-PD-1 therapy, we performed mass cytometry on peripheral blood mononuclear cell (PBMC) samples collected from non-small cell lung cancer (NSCLC) patients prior to treatment with anti-PD-1 therapy (n=26 patients), as well as 10 matched patient samples collected after treatment (**Figures 1A, B**). Detailed characteristics of subjects are described in the Methods section and listed in **Supplementary Table 1**. PBMCs from patients were stained with a comprehensive monocyte-focused CyTOF panel (**Figure 1C**) derived from our study analyzing monocyte heterogeneity in healthy humans (29). To evaluate the interplay between monocytes and T lymphocytes, we added markers for quantifying the frequencies of CD4^+^ and CD8^+^ T cells, including the chemokine receptor CXCR3 (30). To examine the impact of immune checkpoint blockade on the phenotypes of the immune compartment overall, we also incorporated antibodies for PD-1, PD-L1, and PD-L2. We used healthy PBMC samples as spike-in controls and for batch correction of the data using *cydar* (**Supplementary Figure 1A**).

To define major cell types, we performed clustering on 5,359,566 live CD45^+^ cells with all surface markers *via* FlowSOM (20). Classical (cMo), intermediate (iMo), and nonclassical (nMo) monocytes grouped together (**Figure 1D**), and were distinguishable from other blood leukocytes based on both their expression of CD14 and CD16 and their lack of the DC-lineage marker CD1c (**Figure 1E** and **Supplementary Figure 1B**). Examination of the total frequencies of cMo, iMo, and nMo cells revealed no significant changes in these major compartments (**Figure 1F**), which motivated us to focus our studies on subsets of monocytes and monocyte-derived cells.

### Classical Monocytes Exhibit Marked Diversity in NSCLC

**Figure 2:**
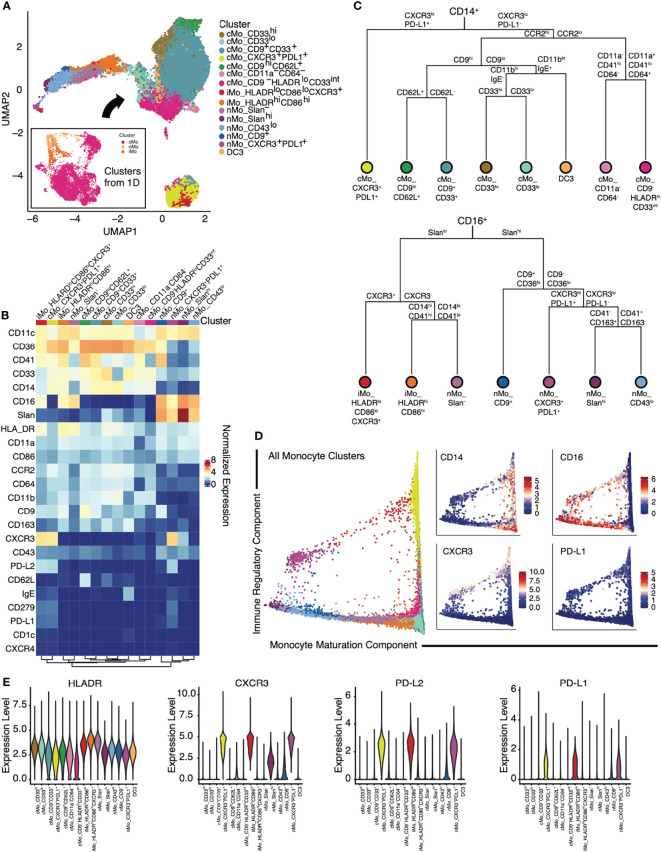
Identification of monocyte subsets in patients with NSCLC using mass cytometry. **(A)** Myeloid clusters from **Figure 1D**, (excluding DC) were subclustered using FlowSOM. A UMAP of the 15 identified monocyte subsets is shown. **(B)** Heat map of inverse hyperbolic sine (arcsinh)-normalized marker expression for each monocyte cluster. **(C)** Dendrograms of the cellular subsets within CD14^+^ (top) or CD16^+^ (bottom) monocytes, based on the Euclidean distance between the normalized expression of each surface marker from **(B)**. **(D)** Trajectory analyses of identified monocytes *via* diffusion map pseudotime identified two components: a monocyte maturation component and an immune regulatory component. Expression of CD14, CD16, CXCR3 and PD-L1 along the same trajectory map are shown in the panels to the right. **(E)** Violin plots of expression of key immune regulatory components (HLADR, CXCR3, PD-L2, and PD-L1) on monocyte subsets.

We thus sub-clustered monocytes (excluding dendritic cells) from the CD45^+^ cells (**Figure 1D**) for all samples (**Figure 2A**). After refining monocyte clusters based on median surface marker expression using consensus clustering, we identified 8 cMo subsets, 2 iMo subsets, and 5 nMo subsets (**Figures 2A–C**; **Supplementary Figures 2A, B**). In particular, we discovered the presence of new nMo subsets (nMo_CD43^lo^, nMo_CD9^+^, and nMo_CXCR3^+^PDL1^+^; **Figures 2A–C**) that we did not observe in PBMCs from healthy humans (29). Although we observed these subsets in all patients, we noted variability in their frequencies amongst individual NSCLC patients (**Supplementary Figure 2C**), similar to what we’ve seen before (29). Nonclassical monocytes displayed enrichment of CD16, CD86, CD11a, and CD11c, but lower levels of CD14, CD11b, CD64, CCR2, and CD36 (**Figure 2B** and **Supplementary Figure 2A**). Notably, nMo clusters expressed less CD33 than most cMo clusters, except for cMo_CD33^lo^. Four nMo subsets expressed Slan, a carbohydrate modification of P-selectin glycoprotein-1 (PSGL-1), and were further distinguished by differential expression of CD9. One nMo cluster was quite high for Slan enrichment (nMo_Slan^hi^). Intermediate monocytes displayed dual expression of classical and nonclassical monocyte proteins including high expression of CD14, CD16, CD36, CD64, CD86, CD11a, CD11b, and CD11c, but lower expression of CCR2 and CD64 (**Figure 2B** and **Supplementary Figure 2A**). This profile is consistent with our previous analysis of healthy human PBMCs demonstrating that exclusion gating based on CCR2, CD36, HLADR, and CD11c improves the purity of intermediate monocytes (31).

Classical monocytes expressed CD14, CCR2, CD36, and CD11b (**Figure 2B** and **Supplementary Figure 2A**), yet certain subsets could be distinguished based on expression of CD33, a siglec that is preferentially expressed on peripheral myeloid cells (32), and the tetraspanin CD9, which is involved in lipid raft clustering. As CD33 is often used to identify cells on the entire monocyte lineage, the presence of CD33^lo^ monocytes indicates that this marker should not be used in isolation. cMo_CD9^-^HLADR^lo^ and cMo_CD9^+^ appeared similar to CD9^+^ and CD9^-^ classical monocyte subsets that we previously reported (29). Two cMo subsets (cMo CD11a^-^CD64^-^ and cMo CD9^-^HLADR^lo^ had lower expression of the activation markers CD64, CD11a, and CD86 (**Figure 2B** and **Supplementary Figure 2A**), suggesting that these are either immature monocyte phenotypes or the newly emigrated monocyte precursors found in COVID-19 (33, 34). Ginhoux’s group recently identified the inflammatory DC3 subset, which expresses CD14, CD163, and binds IgE (35). Based on marker expression analysis, we observe DC3 cells in our NSCLC patient cohorts (**Figures 2A, B** and **Supplementary Figures 2A, B**). A dendrogram showing the relationships amongst all monocytes present in the NSCLC patients is presented (**Figure 2C** and **Supplementary Figure 2B**), highlighting increased levels of diversity amongst cMo compared to nMo.

### Monocyte-Derived Cells Display Global Expression of PD-L1 and CXCR3 in Patients With NSCLC

Trajectory analyses using diffusion maps indicated two trajectory components: a monocyte maturation component and an immune regulatory component (**Figure 2D**). The first diffusion component correlated with monocyte maturing, in concordance with a continuous differentiation trajectory from cMo through iMo and into nMo (36, 37). The second diffusion component correlated with the expression of CXCR3 and PD-L1, distinguishing CXCR3^+^ monocytes from CXCR3^-^ subsets. These data suggest that CXCR3^+^ classical monocytes give rise to CXCR3^+^ nonclassical monocytes in a process parallel to the differentiation of CXCR3^-^ monocytes. To establish that CXCR3^+^ monocytes are single-nucleated cells, we confirmed that all CXCR3^+^ monocyte subsets had a similar signal intensity in the DNA intercalator channel (data not shown) and expressed low levels of lineage markers CD3, CD19, CD66b, CD161, and Siglec-8. Our trajectory analysis indicates that nMo_Slan^-^ monocytes give rise to nMo_Slan^hi^ monocytes (**Figures 2A, D**).

Given the importance of myeloid-specific expression of antigen-presenting molecules in anti-tumoral immunity (38), we examined myeloid expression of surface molecules that modulate adaptive immune responses in our NSCLC cohorts (7). HLADR was differentially expressed between monocyte subsets (**Figure 2E**). As expected, an iMo subset expressed the highest levels of HLADR (31); yet nMo_Slan^-^ also showed very high HLADR expression, suggesting that these monocyte subsets may be involved in antigen presentation. The high expression of HLADR in iMo is consistent with work in mice demonstrating that Ly6C^int^ monocytes express MHCII-associated genes CD74 and H2-aa (36). PD-L1 has been shown to be exclusively expressed by nonclassical monocytes in mice (39). However, we found that PD-L1, together with PD-L2 and their receptor PD-1, were expressed across different monocyte subsets, including cMo and iMo in NSCLC patients (**Figures 2C–E**). Our clustering analysis showed that they formed distinct, separate clusters from other monocytes (yellow island in bottom of UMAP in **Figure 2A**).

### Expression of CD33 by Monocytes Associates With Anti-PD-1 Responsiveness

**Figure 3:**
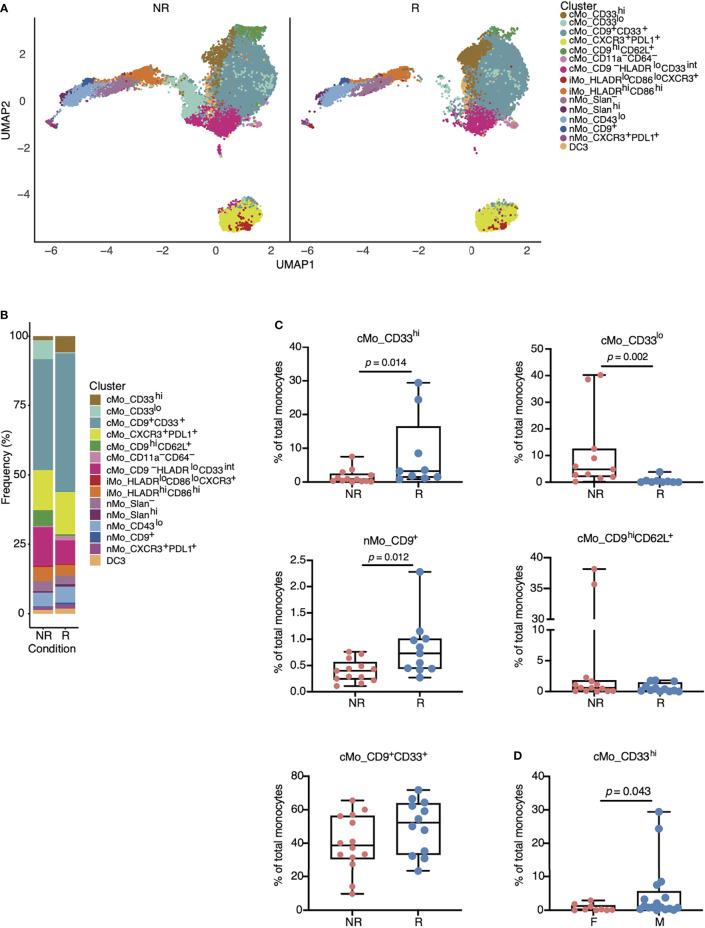
Specific monocyte subsets are associated with responsiveness to anti-PD-1 therapy in patients with NSCLC. **(A)** UMAP from **Figure 2A** split amongst NR versus R to anti-PD-1 treatment. Responders displayed increased numbers of cMo_CD33^hi^ (gold) and nMo_CD9^+^ (dark blue) cells, yet reduced numbers of cMo_CD33^lo^ (aqua) cells. **(B)** Frequencies of each monocyte cluster in PBMCs from NR versus R based on data from **(A)**. **(C)** Boxplots indicating statistically significant changes in cMo_CD33^hi^, nMo_CD9^+^, and cMo_CD33^lo^, and non-significant changes in cMo_CD9^hi^CD33^+^ and cMo_CD9^hi^CD62L^+^. Each dot represents an individual patient. **(D)** Differences in cMo_CD33^hi^ monocyte subset frequencies between males and females. All *p*-values displayed in **(C, D)** were determined by a Mann-Whitney test.

Total CD14^+^ cMo frequencies are predictive of anti-PD-1 responses in melanoma patients (14), but we did not observe this in our cohorts of NSCLC patients (**Figure 1F**). To determine if specific monocyte subsets distinguished non-responders from responders amongst NSCLC patients treated with anti-PD-1, we split our monocyte UMAP between non-responders and responders (**Figure 3A**). We also quantified each monocyte subset amongst all patients (**Figure 3B** and **Supplementary Figure 2C**). Responders displayed significantly reduced frequencies of the cMo_CD33^lo^ subset and significantly increased frequencies of cMo_CD33^hi^ and nMo_CD9^+^ monocytes (**Figure 3C**). cMo_CD33^hi^ cells were significantly higher in responders, and cMo_CD33^lo^ cells were higher in non-responders (**Figure 3C**). There was no significant difference in either cMo_CD9^+^CD33^+^ or cMo_CD9^hi^CD62L^+^ cells between NR and R (**Figure 3C**). cMo_CD33^hi^ cells were significantly higher in males than females (**Figure 3D**); this trend held true for both responders and non-responders (**Supplementary Figure 3**). Males exhibited higher levels of DC3, regardless of ICB responsiveness, but this did not reach statistical significance (**Supplementary Figure 3**).

**Figure 4:**
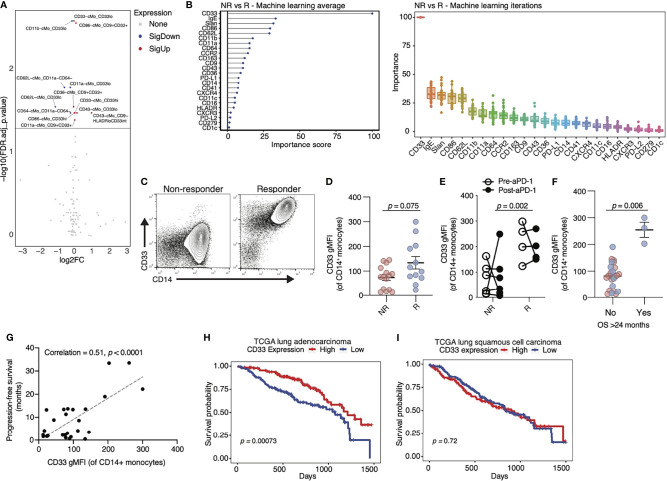
Expression of CD33 by classical monocytes associates with positive responses to anti-PD-1 therapy in cancer. **(A)** Volcano plot of markers on classical monocytes (cMo) that are significantly different in R compared to NR. Red = significantly higher in R vs NR, blue= significantly lower in R vs NR. Significance is defined by an FDR-adjusted *p*-value below 0.05. **(B)** Average (left) and individual (right) importance scores from 20 independent iterations of a random forest machine learning model identify CD33 as the top monocyte marker in distinguishing NR from R. Data are scaled to 0-100. **(C)** Flow cytometry analysis of PBMCs from additional NSCLC patients in our cohorts confirms an increase in CD33 in R versus NR (representative flow plot shown). **(D)** Quantification of geometric mean fluorescent intensity (gMFI) of CD33 on cMo from flow cytometry analysis represented in **(C)**. Each dot represents an individual patient. The *p-*value (0.075) was obtained from a Mann-Whitney test. **(E)** In a paired analysis of monocytes from ten patients pre (open circles) and post (closed circles) anti-PD-1 treatment, patients with R status had higher levels of CD33 gMFI compared to patients with NR status, even at baseline. There were no changes in CD33 gMFI between patients with NR and R status after treatment with anti-PD-1. **(F)** Quantification of CD33 gMFI on cMo by flow cytometry in NSCLC patients grouped by survival (OS) > 24 months. **(G)** Correlation of patient survival with CD33 gMFI of cMo in our NSCLC cohorts. **(H)** Kaplan-Meier curves of CD33 expression (red = high; blue = low) in the lung adenocarcinoma cohort of the TCGA database. **(I)** Kaplan-Meier curves of CD33 expression (red = high; blue = low) in the lung squamous cell carcinoma cohort of the TCGA database. The *p*-values displayed in **(D–F)** were determined by a Mann-Whitney test. The *p*-value displayed in **(G)** were determined by a linear regression. The *p*-values displayed in **(H, I)** were determined by a log-rank test.

To support the importance of CD33 and CD9 in determining anti-PD-1 ICB responses, and isolate other markers of interest in cMo and nMo subsets, we employed linear modeling *via* the *limma* and *diffcyt* packages, which apply high-resolution clustering and Bayesian statistical modeling (40). Consistent with our findings above, CD33 was the marker best able to distinguish responder and non-responder patients (**Figure 4A**). As a second independent analysis method, we utilized machine learning to examine monocyte markers that were most associated with responses to anti-PD-1 in our NSCLC cohorts. The random forest model was trained with the normalized marker expression from randomly selected cells over 20 iterations, and variable importance scores of the markers were calculated and scaled to 100% (41). Using this second method, we again found that CD33 was the highest ranked marker in terms of importance for predicting positive responses to anti-PD-1 (**Figure 4B** and **Supplementary Figure 4A**). Other key markers identified were IgE, Slan, CD86 and CD62L. We validated CD33 expression on cMo in PBMCs from responders and non-responders by flow cytometry (**Figure 4C**). While not statistically significant, the average expression of CD33 was higher in responders compared to non-responders (**Figure 4D**).

We next examined whether anti-PD-1 therapy altered the frequency of CD33^hi^ cMo. We compared baseline (pre-) and post-treatment samples collected from the same patients between 2 and 12 weeks after their first dose of anti-PD-1. Responders had increased cMo_CD33^hi^ levels at baseline, before treatment (**Figure 4E**). We found that anti-PD-1 therapy caused no change in CD33 expression on cMo, as post-treatment levels were similar to pre-treatment levels in patients (**Figure 4E**). To validate the utility of our findings in other cancer types, we analyzed an independent cohort of melanoma patients treated with anti-PD-1. We observed a confirmatory increase in CD33 expression on cMo in responders to anti-PD-1 therapy by flow cytometry (**Supplementary Figure 4B**). As CD33 was by far the most significant marker identified in terms of predicting anti-PD-1 responses, we next examined correlations between CD33 overall survival (OS) in our NSCLC cohorts. CD33 was associated with OS out to >24 months (**Figure 4F**). We also observed a significant correlation between PFS and CD33 expression on cMo in our cohorts (**Figure 4G**). Examination of lung adenocarcinoma data from the TCGA cohort (**Figure 4H**) showed that high CD33 expression was associated with increased survival. In contrast, CD33 expression was not associated with improved survival in lung squamous cell carcinoma data from the TCGA (**Figure 4I**). Survival amongst patients with skin cutaneous melanoma was associated with high CD33 expression in the TCGA cohort (**Supplementary Figure 4C**). These observations suggest that CD33 expression may be strongly associated with anti-PD-1 responses in specific cancer subtypes.

### CD9 and CD86 Expression by Monocytes Correlates With Anti-PD-1 Responder Status

**Figure 5:**
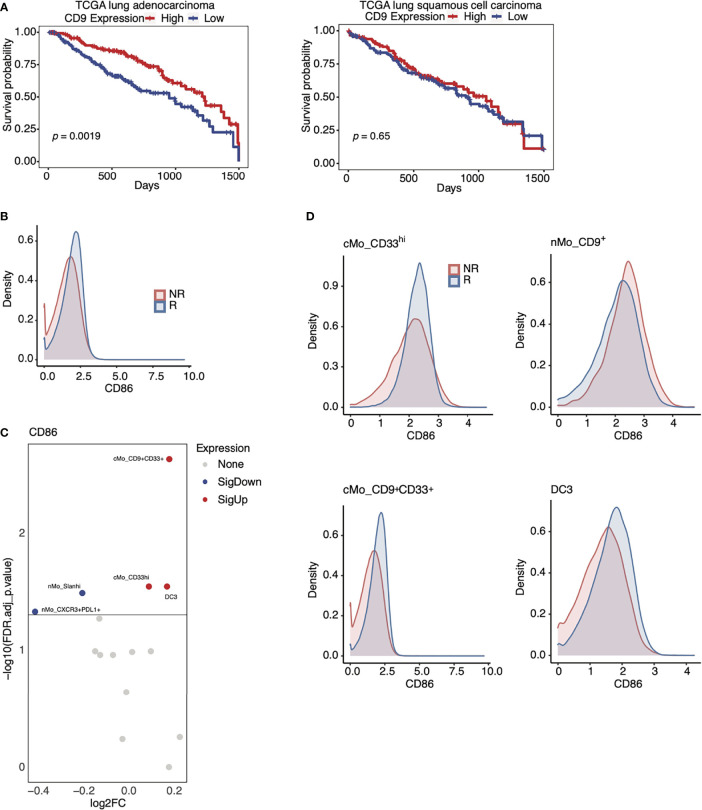
Expression of CD9 and CD86 in monocytes from anti-PD-1 therapy responders. **(A)** Kaplan-Meier curves of CD9 expression in the lung adenocarcinoma cohort (left) and the lung squamous cell carcinoma (right) of the TCGA database (red = high; blue = low). **(B)** Density plot of CD86 expression amongst all monocyte subsets in R (blue) and NR (red) in our NSCLC cohorts. **(C)** Volcano plots showing that CD86 expression on cMo_CD9^+^CD33^+^, cMo_CD33^hi^, and DC3 is associated with positive responses to anti-PD-1 in our NSCLC cohort (red= increased; blue= decreased). **(D)** Density plots showing that CD86 expression amongst key monocyte subsets identified in **Figure 3C** and **(C)** is associated with positive responses to anti-PD-1. The *p*-values displayed in **(A)** were determined by a log-rank test.

We next directed our attention towards CD9 because our findings linked nMo_CD9^+^ monocytes with anti-PD-1 responder status (**Figure 3C**). CD9 was moderately associated with anti-PD-1 responsiveness *via* our machine learning analysis (**Figure 4B** and **Supplementary Figure 4A**). Additionally, CD9 expression was linked to improved survival in patients with lung adenocarcinoma, but not in patients with lung squamous cell carcinoma, from the TCGA cohort (**Figure 5A**). Thus, CD9 may serve as an indicator of anti-PD-1 responsiveness in an NSCLC subtype-dependent manner.

CD86 was enriched in cMo_CD9^+^CD33^+^ monocytes, cMo_CD33^hi^ monocytes, and DC3 cells from responders (**Figure 5C**). CD86 expression was highest in cMo_CD33^hi^ cells, but was also elevated amongst cMo_CD9^+^CD33^+^ monocytes and DC3 (**Figure 5D**) in responders compared to non-responders. Our machine learning analysis highlighted CD86 as another marker associated with responsiveness to ICB therapy (**Figure 4B**), and we found that CD86 expression was higher amongst all monocytes from responders versus non-responders (**Figure 5B**). As CD86 contributes to antigen-presentation, these data imply an increased immune response by these cellular subsets in responders to anti-PD-1 therapy. This model is consistent with the finding that CD86 expression by some monocytes and DCs is linked to benefit from anti-PD-1 therapy in melanoma patients (42). These data also support the notion that CD9 plays a role in determining the anti-PD-1 response, as CD86 expression on nMo_CD9^+^ monocytes, although high, was not higher in responders than non-responders (**Figure 5D**).

### Interactions Between Monocytes and Regulatory T Cells Are Perturbed During NSCLC Progression

**Figure 6:**
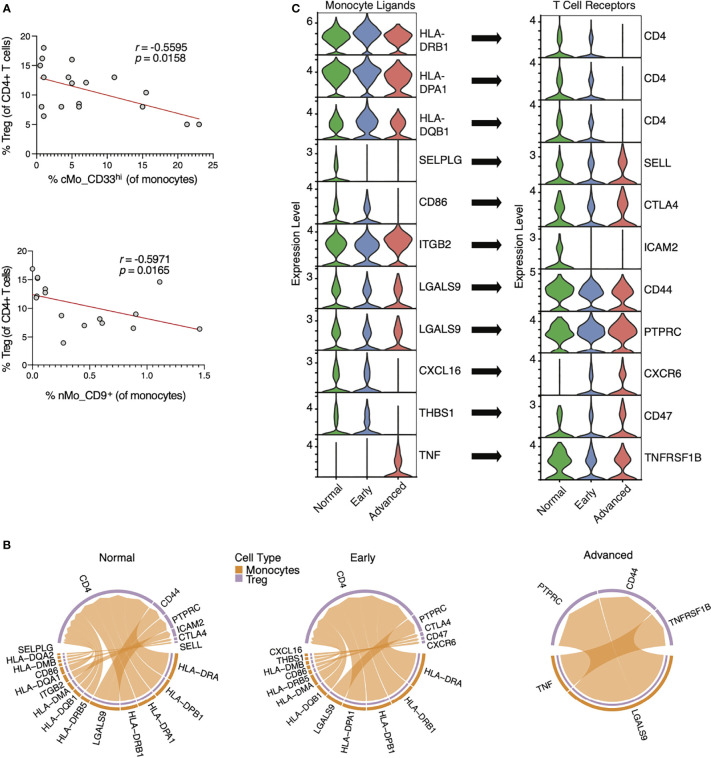
Interactions between monocyte ligands and T_Reg_ cell receptors in lung adenocarcinoma. **(A)** Correlations of cMo_CD33^hi^ and nMo_CD9^+^ monocyte frequencies with T_Reg_ cell frequencies by flow cytometry in the blood of NSCLC patients. **(B)** Chord plots showing probable interactions between ‘sender’ monocytes (orange) and ‘receiver’ T_Reg_ cells (purple) in scRNA-seq data from both healthy patients and patients with early and advanced lung adenocarcinoma (27) using the *CellChat* algorithm. **(C)** Violin plots showing temporal expression of RNA transcripts for monocyte ligands and T_Reg_ receptors identified in **(B)**. Violin plots are ordered to show alignment of monocyte ligands with their cognate T_Reg_ receptors, and arrows indicate the respective ligand:receptor pairings.

To link monocyte subset functions with T cells in NSCLC, we performed flow cytometry for the 3 monocyte subsets shown in **Figure 3C** and regulatory T (CD4^+^FOXP3^+^CD25^+^; T_Reg_) cells within the same patients. T_Reg_ cells are linked to tumor progression *via* their promotion of immune suppression (43), and we thus hypothesized that cMo_CD33^hi^ and nMo_CD9^+^ monocytes would negatively correspond with T_Reg_ frequencies. Both cMo_CD33^hi^ and nMo_CD9^+^ monocyte frequencies were significantly correlated with reduced numbers of T_Reg_ cells in the same NSCLC patients (**Figure 6A**).

We next analyzed a recently published scRNA-seq dataset from patients with lung adenocarcinoma (27) to explore how T cells interact with monocytes during NSCLC. In the Kim data set, scRNA-seq was performed on tumor biopsies from patients with early (Stage I) or advanced (Stage III) LUAD or adjacent normal lung tissues (Normal). We thus used the *CellChat* algorithm to quantitatively evaluate T_Reg_ cells cell and monocyte communication within this scRNA-seq dataset. The number of interactions between monocytes and T_Reg_ cells decreased in early cancer tissue compared to normal lung tissue, and diminished during progression from early to advanced cancer (**Figure 6B**). We also observed an increase in TNF signals from monocytes to T_Reg_ cells in lung tissue of advanced cancer versus healthy lung. Finally, HLA interactions were reduced from early to advanced disease, with loss of CD4 expression in advanced cancers.

PSGL-1 and CD86 signals from monocytes were diminished during cancer progression, suggesting a less immunologically active tumor (**Figure 6C**). Beta 2 integrin interactions with ICAM2 were also reduced, as are galectin9-CD44 interactions, which are important for T cell activation. CXCL16 is a putative biomarker for responses of NSCLC patients to Bevacizumab (anti-VEGF) (44), which is consistent with the reduced CXCL16-CXCR6 interactions during cancer progression herein (**Figures 6B, C**). Together, these data show significant loss of monocyte:T cell interactions during cancer progression that leads to a reduction in anti-tumoral phenotypes over time.

## Discussion

Anti-PD-1 and other ICB therapies have revolutionized cancer treatment (2, 3, 45). Monocytes and monocyte-derived cells consist of multiple subpopulations that uniquely respond to cancer, and thus represent a rich compartment for identifying biomarkers allied with cancer remission and ICB responsiveness. Herein, we used CyTOF mass cytometry to link monocyte heterogeneity with responsiveness to PD-1 blockade in patients with NSCLC. We found that a subset of circulating CD14^+^CD33^+^ monocytes were significantly associated with anti-PD-1 therapy responsiveness in NSCLC patients. We also examined a separate cohort of melanoma patients and found a similar elevation of CD14^+^CD33^+^ monocytes in patients responsive to PD-1 blockade.

Human CD33 is a sialic acid-binding immunoglobulin-like lectin (siglec-3) that stunts myeloid cell activation *via* an immunoreceptor tyrosine-based inhibitory (ITIM) domain (46). CD33 counteracts the scavenger receptor TREM2 in microglia *via* upstream signaling (46, 47). CD33 expression is likewise connected to a myeloid-derived suppressor cell phenotype (48, 49). CD33 is often used as a lineage marker for all monocytes, but our identification of CD33^lo^ monocytes suggests that this strategy is not appropriate. CD33 expression is increased under hypoxic conditions *in vitro*, which concords with increased levels of CD33^+^ cells within tumors (50). Thus, differential expression of CD33 by monocyte populations can drive subset-specific functions during NSCLC progression.

Our data suggest that CD33 expression by human monocytes is predictive of the effectiveness of anti-PD-1 treatment. Alternatively, CD33^+^ monocytes may represent a prognostic factor for NSCLC survival in general (**Figures 4F–G**). We addressed this by testing associations between CD33 expression and survival in patients with either lung adenocarcinoma and lung squamous cell carcinoma. High CD33 expression was associated with a significantly improved survival rate in patients with lung adenocarcinoma from the TCGA dataset (**Figure 4H**). However, we found no significant association between monocyte CD33 expression and survival in patients with lung squamous cell carcinoma within the TCGA cohort. Additionally, we found that monocyte CD33 expression was higher in melanoma patients that responded to anti-PD-1 (**Supplementary Figure 4B**), and CD33 was also associated with better survival in melanoma patients within the TCGA dataset (**Supplementary 4C**). Thus, CD33 enriched on monocytes is predictive of positive anti-PD1 responses in both NSCLC and melanoma. However, we were not able to fully determine the prognostic value of CD33 in cancer subtypes herein, given the limited nature of our clinical data. Future work will test the predictive and prognostic values of CD33 in NSCLC subtypes.

To date, no one has examined differences in the nMo compartment between males and females. Here, we found that cMo_CD33^hi^ levels were lower in the blood of females than males with NSCLC (**Figure 3C**). As females have been shown to have reduced responsiveness to anti-PD-1 therapy on average (51), cMo_CD33^hi^ levels in females could be a strong indicator of female ICB responsiveness. We also found that a nonclassical monocyte nMo_CD9^+^ subset was associated with positive responses to anti-PD-1 treatment. CD9 is a tetraspanin that sequesters receptors such as CD36, MHCII, and TLR4 within lipid microdomains on the plasma membrane (52, 53). Trafficking of MHC to the cell surface is thought to be CD9-dependent (54, 55). These cells also expressed higher levels of the TLR4 co-receptor CD14 than other nMo subsets, but this enrichment was lower than that of classical monocytes (**Figure 2B** and **Supplementary Figure 2A**). Murine macrophages lacking CD9 cannot phagocytose ligands such as oxidized LDL due to impaired CD36 activity (52). CD9 also negatively regulates LPS signaling *via* control of CD14 and TLR4 clustering on membrane lipid rafts (56). Although they composed a rare monocyte subset, nMo_CD9^+^ cells were inversely correlated with T_Reg_ frequencies in our matched samples from patients with NSCLC. Levels of nMo_CD9^+^ cells were higher in both male and female patients that responded to ICB compared to non-responders (**Supplementary Figure 3B**).

DC3 cells are thought to represent an inflammatory CD14^+^CD163^+^ subset derived from DC2 cells, but the details of their ontogeny have yet to be fully characterized. We previously reported the identification of a IgE^+^CD14^+^CD163^+^ cMo subset in healthy human blood *via* CyTOF (29), but which likely represent either DC2 or DC3 cells (35). In our current study, the DC3 subset clustered with monocytes and not with DCs, and presented with low expression of the typical human DC marker CD1c. These data suggest that at least some DC3 cells arise from the monocyte pool. While lineage tracing studies would be necessary to confirm this lineage model, it is consistent with our trajectory analyses. Peripheral DC3 frequencies were higher in males than females, but they were not associated with responses to PD-1 blockade (**Supplementary Figure 3B**).

Merad and colleagues recently identified an immunoregulatory DC that expresses PD-L2 and CCR7, in both tumor-bearing mice and humans with NSCLC (38). These mregDC are less immunogenic than other DC subsets and are likely derived from DC1 and DC2. In our current study, we identified ‘immunoregulatory’ monocytes (IR-Mo) from NSCLC patients that expressed CXCR3, PD-L1, and PD-L2. Thus, our data indicate that IR-Mo are distinguishable from mregDC, as IR-Mo appear to be derived from the monocyte pool. While recent work has shown that PD-L1 is exclusively expressed in nonclassical monocytes in mice (39), we found PD-L1 and PD-L2 to be expressed amongst all conventional monocyte pools. Induction of PD-L1 in monocytes and DC occurs *via* IFNg, and PD-L1 exerts tolerogenic, immunosuppressive functions to support tumor growth (57). Importantly, several clinical trials have reported that high PD-L1 expression is linked with better response to PD-1 blockade and longer survival (45). Thus, we hypothesize that while these cells are likely pro-tumoral in nature, their presence may indicate patients likely to benefit from ICB therapy. CXCR3 expression on monocytes has been shown to promote lung metastasis in a mouse model of metastatic melanoma (58), and like PD-L1, is induced by interferon signaling (59). Thus, IR-Mo cells likely arise in response to NSCLC progression.

We found that both cMo_CD33^hi^ and nMo_CD9^+^ cells were negatively correlated with T_Reg_ cells in blood samples from NSCLC patients. We therefore applied the *CellChat* algorithm (28) to a published lung adenocarcinoma scRNA-seq dataset (27), in order to use the expression of cognate surface molecules as a readout for cell-to-cell communication within this dataset. Our analysis revealed a large reduction in the number of purported immunogenic signals from monocytes to T cells. These data illustrate the importance of cell:cell interactions in the TME and how such interactions lose their anti-tumoral potency during cancer progression. Whether these signals are common across multiple cancer types is not known, but several of these receptor-ligand pairs (CXCR6-CXCL16, CTLA-4-CD86, LGALs9-CD44/PTPRC) have been shown to play roles in cancer modulation (60–62).

A limitation of this study is our focus on NSCLC patients who received a single therapy, which is no longer the standard of care (45, 63, 64). However, as anti-PD-1 is well utilized in combination therapy, finding markers that dictate positive responses to anti-PD-1 remains highly useful. While CD33 levels can be readily detected using an ELISA or flow cytometry, whether this marker predicts responses to other ICB treatments is unknown. Future studies will be aimed at understanding the functions of these cellular subsets in the control of cancer.

## Data Availability Statement

The original raw CyTOF files are available upon request. Inquiries can be directed to the corresponding author.

## Ethics Statement

The studies involving human participants were reviewed and approved by and IRB at LJI, IMIM, and UCSD. The participants provided their written and informed consent to participate in this study.

## Author Contributions

CO performed the experiments and analyzed data. AA performed bioinformatics and statistical analyses. DA wrote the manuscript, assisted with data analysis, and prepared the figures. DB assisted with organizing the manuscript and provided intellectual input. NG assisted in the preparation of tables and editing of the manuscript. MW and EA provided human patient samples from IMIM and assisted edited the manuscript. SP provided human patient samples from the UCSD Moores Cancer Center for this study and assisted with manuscript edits. CO edited the manuscript and helped with interpreting clinical data. HD provided intellectual input and data analysis. CH directed and supervised the study and assisted with manuscript editing and organization. All authors contributed to the article and approved the submitted version.

## Funding

The CyTOF Helios at LJI was acquired through NIH Shared Instrumentation Grant S10 OD018499. This work was supported by NIH U01 CA224766, R01 CA202987, and P01 HL136275 (all to CH). This work was also supported by ISCIII PI16/00591 and FIS-ISCIII PI19/00003 (to EA), the Whittaker iCure Foundation (to CO), and by the Gerry and Bill Cowlin Family Foundation (to SP).

## Conflict of Interest

The authors declare that the research was conducted in the absence of any commercial or financial relationships that could be construed as a potential conflict of interest.

## Publisher’s Note

All claims expressed in this article are solely those of the authors and do not necessarily represent those of their affiliated organizations, or those of the publisher, the editors and the reviewers. Any product that may be evaluated in this article, or claim that may be made by its manufacturer, is not guaranteed or endorsed by the publisher.

## Supplementary Material

Supplementary Figure 1 Batch correction of mass cytometry data and identification of CD45^+^ subsets in PBMCs from patients with NSCLC. **(A)** Batch correction of mass cytometry data by *cydar*; corrections for CD33 and CD14 are presented as examples. **(B)** Heat map of scaled marker expression in each major immune cell type identified in **Figures 1D, E**.

Supplementary Figure 2 Identification of monocyte subsets in NSCLC patients. **(A)** Heat map of scaled median marker expression for monocytes identified in **Figures 2A, B**. **(B)** Normalized heat maps amongst either CD14^+^ or CD16^+^ monocytes used to make the dendrograms shown in **Figure 2C**. **(C)** Frequencies of all monocyte clusters from **Figure 2A** in individual patients. Top panel shows all monocytes. Nonclassical monocyte frequencies are enlarged for detail in the bottom panel.

Supplementary Figure 3 Sex differences in monocyte frequencies. Frequencies of cMo_CD33^hi^, DC3, and nMo_CD9^+^ monocytes between males and females and NR versus R. These data complement those shown in **Figure 3**.

Supplementary Figure 4 Random forest machine learning analysis of monocyte markers. **(A)** Raw data from random forest machine learning analysis. Complements scaled data in **Figure 4B**. **(B)** In a separate cohort of melanoma patients treated with anti-PD-1, R showed increased CD33 expression on cMo compared to NR. **(C)** Kaplan-Meier curves of CD33 expression (red = high; blue = low) in the skin cutaneous melanoma cohort of the TCGA database. The *p*-value displayed in **Supplementary Figure 4B** was determined by a Mann-Whitney test. The *p*-value displayed in **Supplementary Figure 4C** was determined by a log-rank test.

Supplementary Table 1 NSCLC patient characteristics. De-identified patient numbers, Diagnosis, Treatment, Age, Sex, Progression Free Survival (PFS) length, and Cohort Site are provided. Red = non-responders, Blue = responders.
