# Supplementary figures and images for: CD33 Expression on Peripheral Blood Monocytes Predicts Efficacy of Anti-PD-1 Immunotherapy Against Non-Small Cell Lung Cancer
Source: Front Immunol. 2022 Apr 14;13:842653. doi: 10.3389/fimmu.2022.842653 (PMC9046782; doi:10.3389/fimmu.2022.842653)

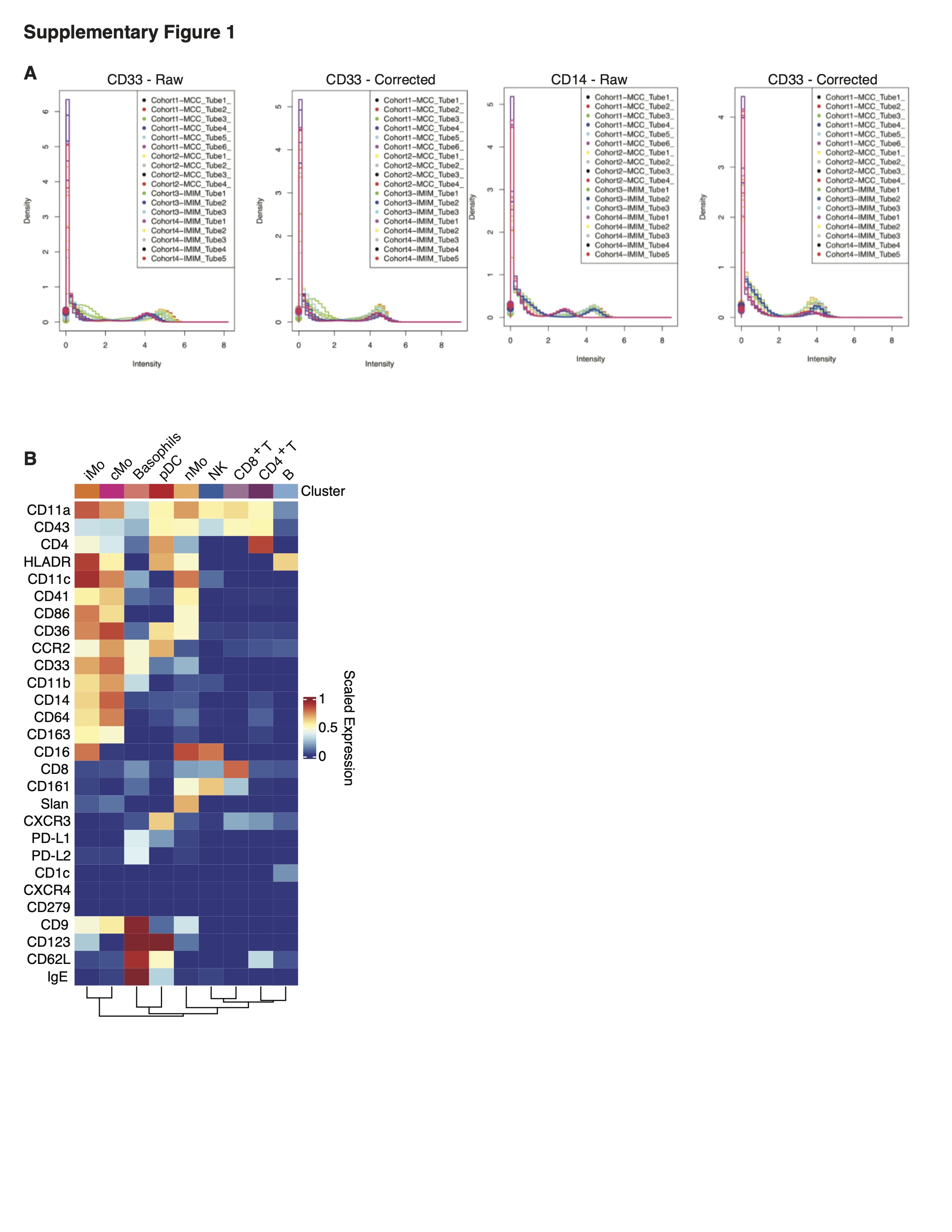

Supplement: Supplementary Figure 1 — Batch correction of mass cytometry data and identification of CD45+ subsets in PBMCs from patients with NSCLC. (A) Batch correction of mass cytometry data by cydar; corrections for CD33 and CD14 are presented as examples. (B) Heat map of scaled marker expression in each major immune cell type identified in Figures 1D, E . [file Image_1.jpeg]

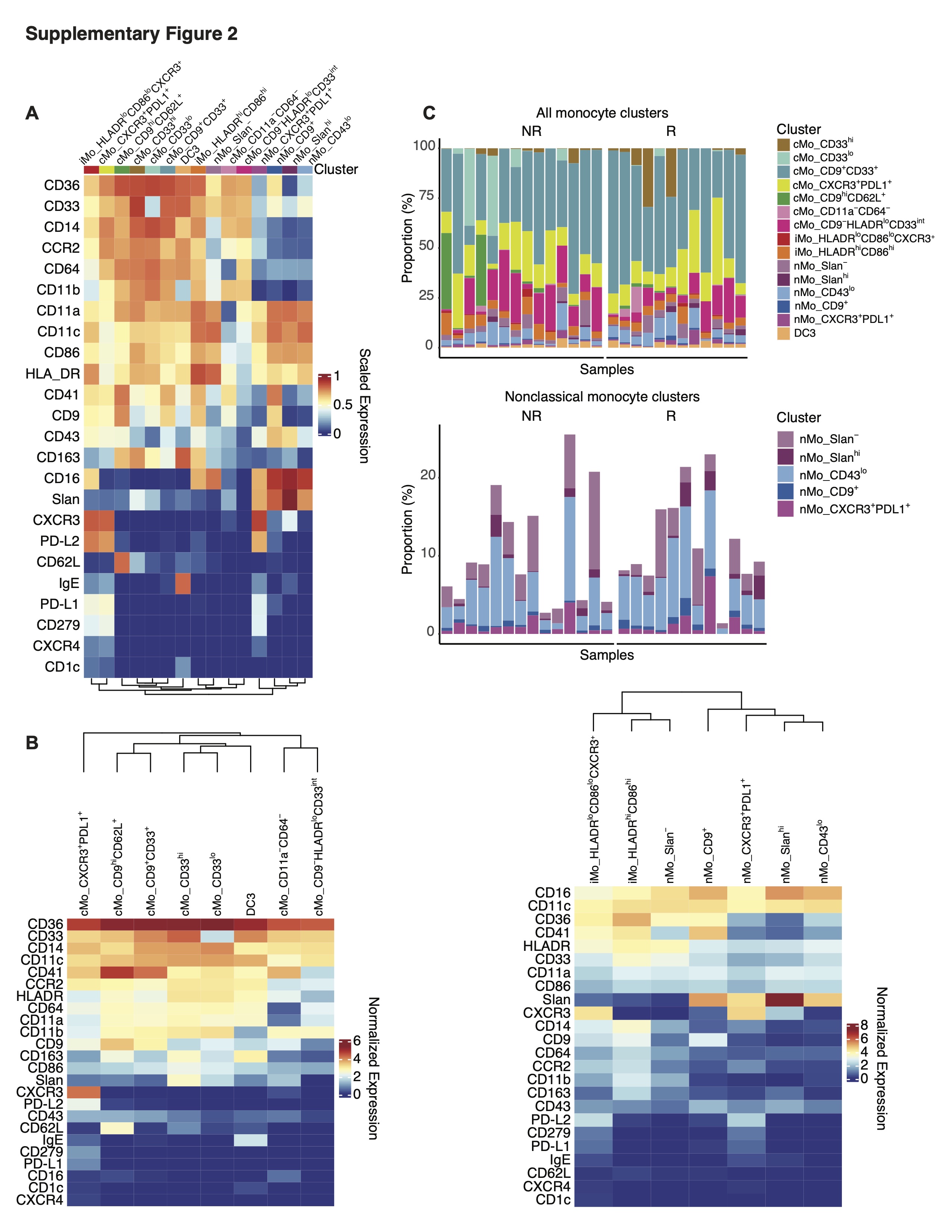

Supplement: Supplementary Figure 2 — Identification of monocyte subsets in NSCLC patients. (A) Heat map of scaled median marker expression for monocytes identified in Figures 2A, B . (B) Normalized heat maps amongst either CD14+ or CD16+ monocytes used to make the dendrograms shown in Figure 2C . (C) Frequencies of all monocyte clusters from Figure 2A in individual patients. Top panel shows all monocytes. Nonclassical monocyte frequencies are enlarged for detail in the bottom panel. [file Image_2.jpeg]

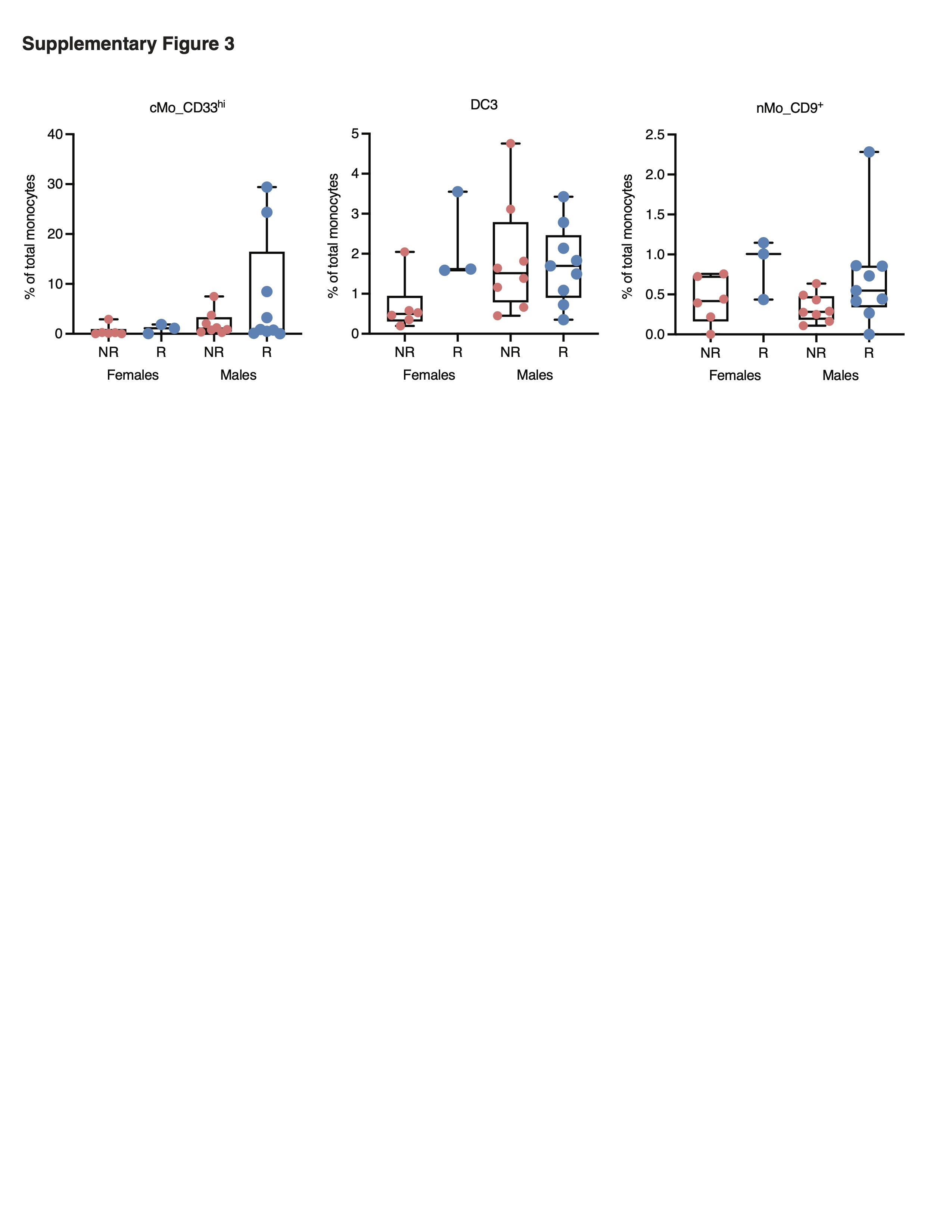

Supplement: Supplementary Figure 3 — Sex differences in monocyte frequencies. Frequencies of cMo_CD33hi, DC3, and nMo_CD9+ monocytes between males and females and NR versus R. These data complement those shown in Figure 3 . [file Image_3.jpeg]

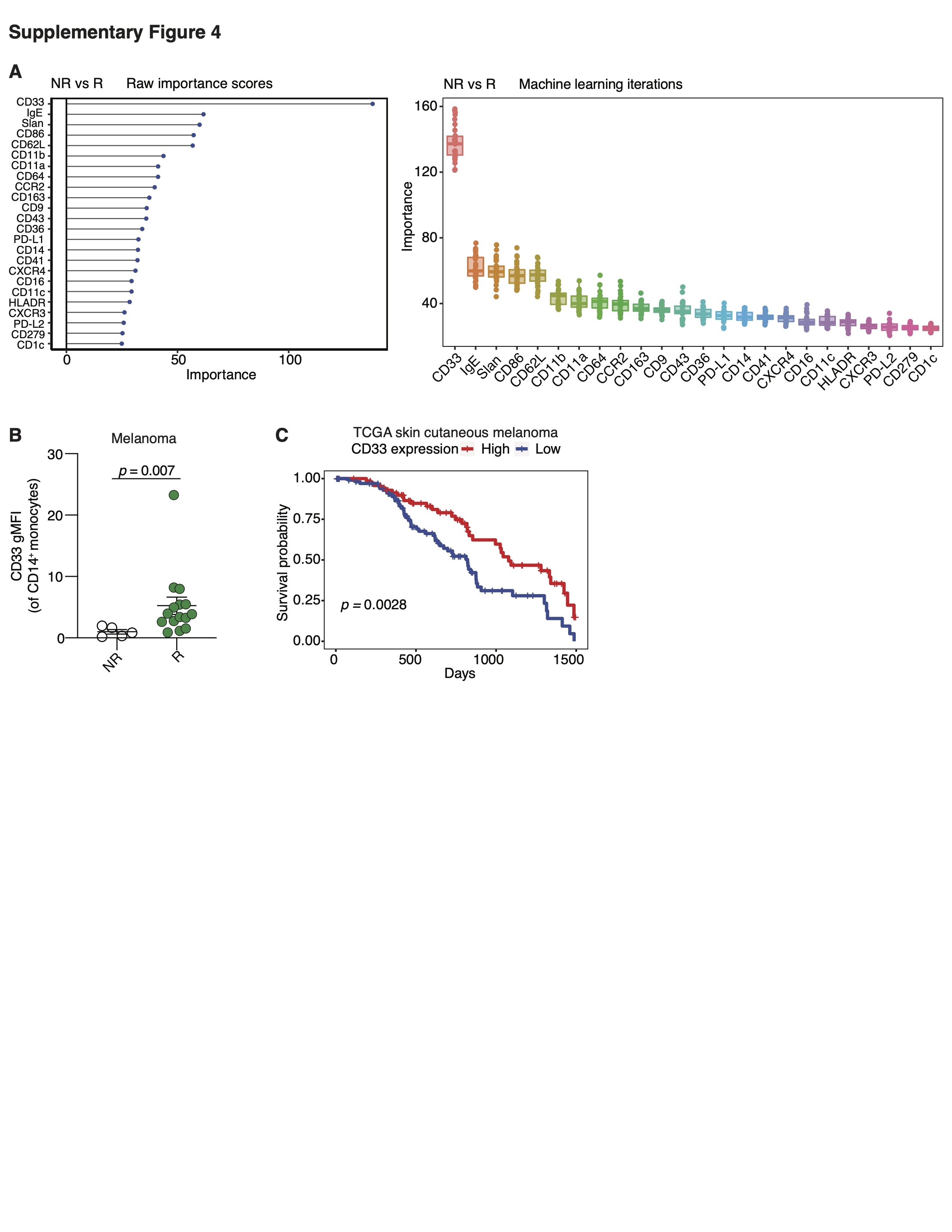

Supplement: Supplementary Figure 4 — Random forest machine learning analysis of monocyte markers. (A) Raw data from random forest machine learning analysis. Complements scaled data in Figure 4B . (B) In a separate cohort of melanoma patients treated with anti-PD-1, R showed increased CD33 expression on cMo compared to NR. (C) Kaplan-Meier curves of CD33 expression (red = high; blue = low) in the skin cutaneous melanoma cohort of the TCGA database. The p-value displayed in Supplementary Figure 4B was determined by a Mann-Whitney test. The p-value displayed in Supplementary Figure 4C was determined by a log-rank test. [file Image_4.jpeg]
