# Supplementary material for: CD33 Expression on Peripheral Blood Monocytes Predicts Efficacy of Anti-PD-1 Immunotherapy Against Non-Small Cell Lung Cancer
Source: Front Immunol. 2022 Apr 14;13:842653. doi: 10.3389/fimmu.2022.842653 (PMC9046782; doi:10.3389/fimmu.2022.842653)
Supplement: Supplementary Table 1 — NSCLC patient characteristics. De-identified patient numbers, Diagnosis, Treatment, Age, Sex, Progression Free Survival (PFS) length, and Cohort Site are provided. Red = non-responders, Blue = responders. [file Table_1.pdf]

Supplementary Table 1

ID	Response	Diagnosis	Treatment	Age/ Sex	PFS (months)	Site
001	NR	Stage IV	Nivolumab	71/F	3.02	MCC
002	NR	Stage III	Nivolumab	64/F	1.87	MCC
003	NR	Stage IV	Nivolumab	64/F	1.58	MCC
004	NR	Stage IV	Nivolumab	46/F	4.10	MCC
005	NR	Stage IV	Nivolumab	83/M	2.23	MCC
006	NR	Stage IV	Nivolumab	80/F	-	MCC
007	NR	Stage IIIB	Nivolumab	72/M	4.10	IMIM
008	NR	Stage IVA	Nivolumab	76/M	1.00	IMIM
009	NR	Stage IVB	Nivolumab	66/M	0.60	IMIM
010	NR	Stage IVA	Nivolumab	72/F	2.20	IMIM
011	NR	Stage IIB	Pembrolizumab	58/M	1.60	IMIM
012	NR	Stage IIIB	Pembrolizumab	67/M	1.40	IMIM
013	NR	Stage IVB	Pembrolizumab	80/M	3.50	IMIM
014	NR	Stage IVB	Pembrolizumab	77/M	0.50	IMIM
015	R	Stage IV	Nivolumab	64/F	22.20	MCC
016	R	Stage III	Nivolumab	46/F	33.53	MCC
017	R	Stage IV	Nivolumab	79/M	33.43	MCC
018	R	Stage IV	Pembrolizumab	84/M	8.57	MCC
019	R	Stage IVB	Nivolumab	59/M	13.2	IMIM
020	R	Stage IIB	Nivolumab	82/M	19.0	IMIM
021	R	Stage IV	Nivolumab	62/M	13.5	IMIM
022	R	Stage IA	Pembrolizumab	69/F	13.3	IMIM
023	R	Stage IV	Nivolumab	65/M	13.4	IMIM
024	R	Stage IVB	Nivolumab	71/M	8.8	IMIM
025	R	Stage IVB	Pembrolizumab	70/M	13.7	IMIM
026	R	Stage IIB	Pembrolizumab	74/M	11.3	IMIM
